# Comparison of National Early Warning Score 2 (NEWS2) and Quick Sequential Organ Failure Assessment (qSOFA) for Predicting In-Hospital Mortality in Patients With Sepsis

**DOI:** 10.7759/cureus.108080

**Published:** 2026-05-01

**Authors:** Arjun V, Sajit Varghese, Rose Merin Joseph

**Affiliations:** 1 Department of General Medicine, Pushpagiri Institute of Medical Sciences & Research Centre, Thiruvalla, IND

**Keywords:** inotropes, mechanical ventilation, mortality, news2, qsofa

## Abstract

Background and objective: Sepsis is a major cause of morbidity and mortality worldwide, requiring early recognition and risk stratification to improve outcomes. Clinical scoring systems such as the quick Sequential Organ Failure Assessment (qSOFA) and National Early Warning Score 2 (NEWS2) are commonly used for bedside assessment. While qSOFA was initially proposed as a rapid screening tool, it is no longer favored for sepsis screening due to its limited sensitivity and is now primarily used for prognostication. In contrast, NEWS2 is widely used for early detection of clinical deterioration and risk stratification. This study aimed to compare the predictive performance of qSOFA and NEWS2 for in-hospital mortality in patients admitted with sepsis in a tertiary care center.

Methodology: This 18-month prospective observational study was conducted at Pushpagiri Medical College Hospital, Thiruvalla, Kerala, India. Consecutive sampling was used to include adult patients with sepsis admitted to the medical intensive care unit (ICU). A total of 155 eligible patients were enrolled. Baseline demographic data, clinical characteristics, comorbidities, and laboratory parameters were recorded at admission. Patients were stratified into high- and low-risk groups based on qSOFA and NEWS2 scores and followed during their hospital stay. In-hospital mortality was the primary outcome, while prolonged ICU stay, need for mechanical ventilation, and requirement for vasopressor or inotropic support were secondary outcomes. Statistical analysis was performed using IBM SPSS Statistics for Windows, Version 20.0 (IBM Corp., Armonk, NY, USA). The chi-square test was used to assess associations, and receiver operating characteristic (ROC) curves were constructed to evaluate predictive performance.

Results: Among the 155 patients, the mean age was 65.6 ± 14.6 years; 82 (53%) were men, and 73 (47%) were women. Within seven days of admission, 29 patients died. Vasopressor support was required in 119 patients, and 66 patients required mechanical ventilation. NEWS2 demonstrated superior predictive performance compared with qSOFA, with higher specificity, negative predictive value, and area under the ROC curve (AUC) across all outcomes. NEWS2 performed better in predicting in-hospital mortality (AUC 0.949), vasopressor requirement (AUC 0.911), need for mechanical ventilation (AUC 0.883), and prolonged ICU stay (AUC 0.707).

Conclusion: This study demonstrated that NEWS2 is superior to qSOFA for predicting clinically relevant outcomes in patients with sepsis, including in-hospital mortality, prolonged ICU stay, mechanical ventilation, and vasopressor support. Its use may facilitate earlier risk stratification and timely clinical intervention. However, as a single-center study, the generalizability of the findings is limited, and further multicenter studies are warranted.

## Introduction

In emergency departments and intensive care units (ICUs), sepsis is a common and possibly dangerous condition [1]. Organ failure and systemic inflammation result from a dysregulated host response to infection [2]. Significant mortality, morbidity, and long-lasting socioeconomic effects are linked to it [3].

Every year, almost 49 million people worldwide suffer from sepsis, which results in nearly 11 million deaths, nearly 20% of all deaths worldwide [4]. Sepsis continues to be a serious public health concern despite a drop in overall death rates; reported mortality rates for those affected are close to 25% [5]. Improving results requires early detection, risk assessment, and prompt action. Different clinical symptoms and the absence of a definitive diagnostic test complicate the diagnosis and prognosis of sepsis [6-8].

The Sepsis-3 work group, established in 2016 by the Society of Critical Care Medicine and the European Society of Intensive Care Medicine, redefined sepsis as a potentially fatal organ dysfunction resulting from a dysregulated host response to infection. Organ dysfunction was defined as an abrupt increase of two or more points in the Sequential Organ Failure Assessment (SOFA) score [9] attributed to infection. To quickly identify high-risk patients at the bedside, the quick SOFA (qSOFA) score was developed [10].

Systolic blood pressure ≤100 mmHg, respiration rate ≥22 breaths per minute, and altered mental status (Glasgow Coma Scale <15) are the minimum requirements for a positive qSOFA score [11]. Although qSOFA is a bedside screening tool, its predictive accuracy for unfavorable outcomes has varied across studies, with reported sensitivity ranging from 50% to 70% and specificity from 70% to 90% for predicting mortality [12].

To standardize the early detection of clinical deterioration, the Royal College of Physicians created the National Early Warning Score (NEWS) in 2012 [13]. It was then called NEWS2 in 2017. Heart rate, oxygen saturation, additional oxygen requirement, respiratory rate, temperature, systolic blood pressure, and level of consciousness are the seven physiological indicators included in NEWS2 [13].

Early detection of high-risk patients enables timely escalation of care and may reduce mortality [8]. When predicting adverse outcomes, NEWS2 has demonstrated higher predictive accuracy than qSOFA in several studies, with reported area under the receiver operating characteristic curve (AUROC) values ranging from 0.77 to 0.89 for mortality prediction, compared with 0.69 to 0.76 for qSOFA [14].

Sepsis is still the leading cause of death in non-coronary ICUs in India; recorded hospital mortality rates are close to 60% [15]. However, there is a dearth of information comparing NEWS2 with qSOFA for sepsis outcome prediction in Indian populations. The purpose of this study was to compare the predictive performance of qSOFA and NEWS2 for in-hospital mortality in patients admitted with sepsis in a tertiary care center.

## Materials and methods

Study design and setting

This single-center observational study was conducted at Pushpagiri Medical College Hospital, Thiruvalla, Kerala, India, over 18 months. Patients who met the predefined inclusion and exclusion criteria were enrolled using a consecutive sampling technique. This was a prospective, observational cohort study in which patients were followed from ICU admission until discharge or in-hospital death.

Sample size

The sample size was calculated assuming a sensitivity of 83.21%, specificity of 57.44%, and a mortality prevalence of 35.12% (adverse outcome) from a reference study [14], with a relative precision of 10% and an alpha error of 10%. The minimum required sample size was estimated to be 98 patients using a standard diagnostic test evaluation approach, and the calculation was performed using an online sample size calculator. Assuming a relative precision of 10% and an alpha error of 10%, the required sample size for sensitivity (NSn) was calculated using the formula:

\[ N_{Sn} = \frac{(Z_{1-\alpha/2})^2 \times Sn \times (1 - Sn)}{d^2} \]

Where

\[ Sn = \text{Sensitivity} = 83.21\% = 0.8321 \]

\[ d = \text{Relative precision of } Sn = 10\% = 0.1 \]

Thus,

\[ N_{Sn} = \frac{(Z_{1-\alpha/2})^2 \times 0.8321 \times (1 - 0.8321)}{(0.1)^2} = 98 \]

Accordingly, the required sample size was 98 patients.

Inclusion and exclusion criteria

Patients aged ≥18 years admitted to the medical ICU with a clinical diagnosis of sepsis according to Sepsis-3 criteria [2] were included after obtaining informed consent from the patient or legally authorized representative. Patients with known immunocompromised states (including malignancy, chemotherapy, or immunotherapy), pregnant women, individuals with recent trauma, and those unwilling or unable to provide consent were excluded.

Data collection method

Demographic information, clinical history, physical examination findings, and laboratory results were collected at admission using a pre-designed proforma. All data were collected prospectively at the time of ICU admission by the principal investigator. The qSOFA and NEWS2 scores were calculated once at admission using initial clinical parameters. Each patient’s quick qSOFA and NEWS2 were calculated at admission and used to classify patients into high- and low-risk groups. Patients were then followed throughout their hospital stay until discharge or death to record outcomes. Data collection was conducted by the principal investigator in strict confidence. Patient history, clinical examination, and hospital records were reviewed, and an investigator-administered form was used to record and organize all study data systematically.

Measurement of study variables

The qSOFA score was calculated using three clinical parameters: respiratory rate ≥22/min, systolic blood pressure ≤100 mmHg, and altered mental status (Glasgow Coma Scale <15), each assigned 1 point, with a total score ranging from 0 to 3. A score of ≥2 was considered high risk. The NEWS2 score was calculated using seven physiological parameters: respiratory rate, oxygen saturation, use of supplemental oxygen, systolic blood pressure, heart rate, level of consciousness, and temperature, each assigned a weighted score according to standard guidelines, with total scores ranging from 0 to 20. A NEWS2 score of ≥5 was considered high risk. The categorization into high- and low-risk groups was based on previously validated cutoff values reported in the literature.

Outcome measurement

The study aimed to compare the predictive accuracy of qSOFA and NEWS2 scores for adverse outcomes in patients with sepsis. The findings are intended to assist clinicians in rapid bedside risk stratification and early management decisions. A scoring system with greater sensitivity for poor outcomes could guide timely interventions and inform future updates to sepsis management guidelines on the routine use of predictive scoring tools. The primary outcome was in-hospital mortality, while secondary outcomes included the requirement for vasopressor or inotropic support, need for mechanical ventilation, and prolonged ICU stay.

Statistical analysis

Collected data were entered into Microsoft Excel (Microsoft, Redmond, WA, USA) and analyzed using IBM^®^ SPSS^®^ version 20 (IBM Corp., Armonk, NY, USA). Continuous variables were presented as mean ± standard deviation, while categorical variables were expressed as frequencies and percentages. Associations between variables were evaluated using the chi-square test. The diagnostic performance of qSOFA and NEWS2 was assessed using 2×2 contingency tables based on predefined cutoffs (qSOFA ≥2 and NEWS2 ≥5), with patients classified as test-positive or test-negative. True positives, true negatives, false positives, and false negatives were identified accordingly and used to calculate sensitivity, specificity, positive predictive value, negative predictive value, and diagnostic accuracy with 95% confidence intervals. Receiver operating characteristic (ROC) curves were constructed to evaluate discriminative ability, and the area under the curve (AUC) was calculated. The predefined cutoff values were used for primary analysis, while ROC curves were used to assess overall performance across different thresholds.

## Results

Among 155 patients, the mean age was 65.6 ± 14.61 years. A total of 82 (53.0%) patients were men. Comorbid conditions were common, with 105 (67.8%) patients having type 2 diabetes and 59 (38.3%) having hypertension. The most frequent sources of infection were respiratory in 56 (36.1%) patients and urological in 45 (28.9%) patients, followed by gastrointestinal in 22 (13.9%) patients and multi-system involvement in 10 (6.7%) patients (Table 1).

**Table 1:** Demographic and clinical characteristics of patients

Variable	Number of patients (total number N = 155)
Age (in years)	65.6 ± 14.61
Sex	
Male	82 (53%)
Female	73 (47%)
Comorbidity	
Type 2 diabetes mellitus	105 (67.8%)
Hypertension	59 (38.3%)
Dyslipidemia	44 (28.3%)
Coronary artery disease	25 (16.1%)
Chronic obstructive pulmonary disease	11 (7.2%)
Other	36 (23.3%)
Source of infection	
Cardiology	1 (0.6%)
Endocrinology	4 (2.8%)
Gastrointestinal	22 (13.9%)
Multi-system	10 (6.7%)
Nephrology	5 (3.3%)
Neurology	3 (1.7%)
Respiratory	56 (36.1%)
Skin and soft tissue	9 (6.1%)
Urology	45 (28.9%)

**Figure 1:**
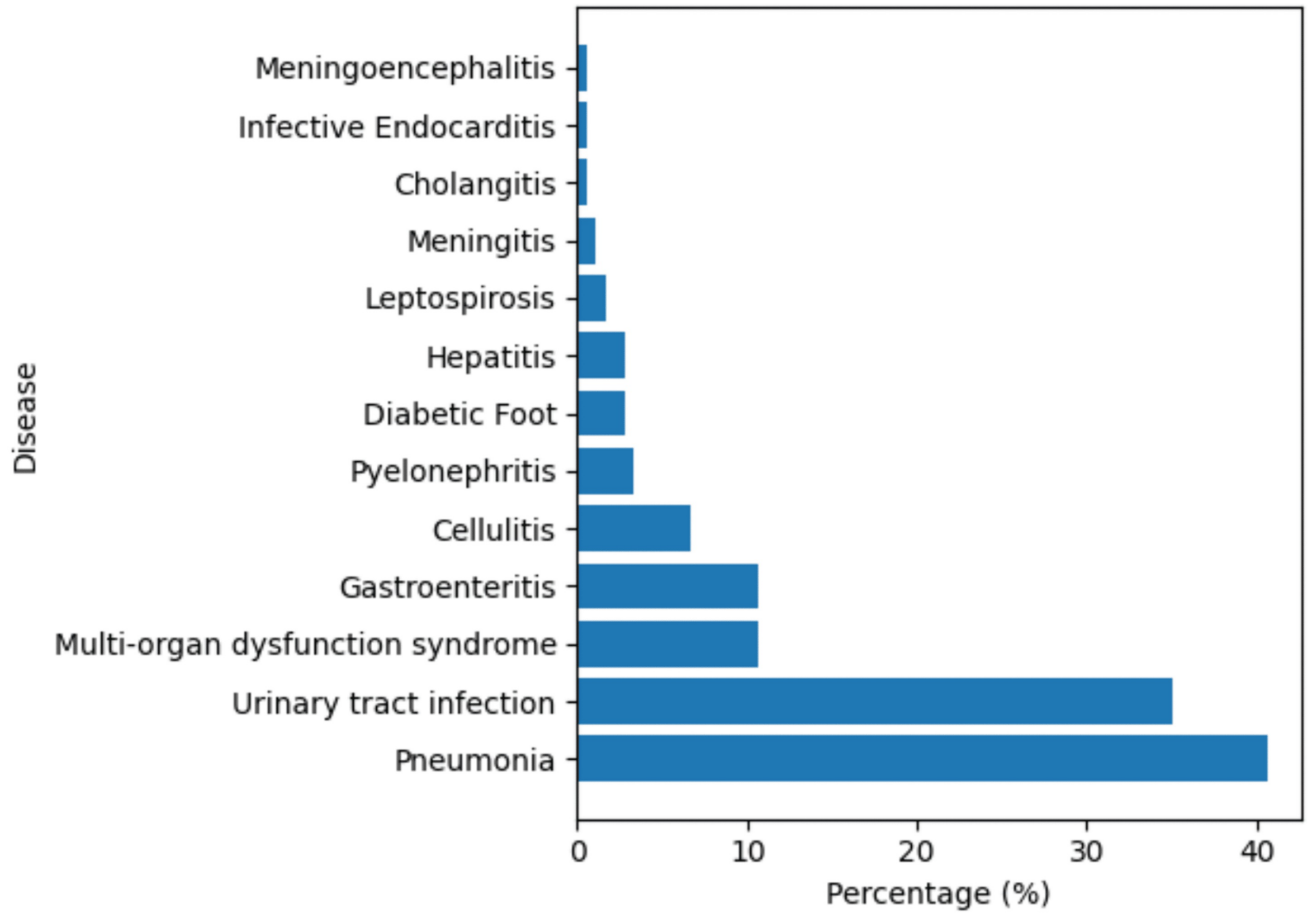
Distribution of primary infectious diagnoses among patients with sepsis

 Among the study population, pneumonia was the most common disease, affecting 73 (40.6%) patients, followed by urinary tract infection, which affected 63 (35%). Multi-organ dysfunction syndrome and gastroenteritis were each seen in 19 (10.6%), cellulitis in 12 (6.7%), pyelonephritis in 6 (3.3%), diabetic foot and hepatitis each in 5 (2.8%), leptospirosis in 3 (1.7%), meningitis in 2 (1.1%), and cholangitis, infective endocarditis, and meningoencephalitis each in 1 (0.6%) (Figure 1).

Table 2 shows the association between qSOFA and NEWS2 scores and clinical outcomes in 155 patients. The first seven-day mortality occurred in 29 patients, all of whom had qSOFA ≥2 and NEWS2 ≥5. Among survivors (N=126), 110 (87.3%) had qSOFA ≥2 and 113 (89.6%) had NEWS2 ≥5. Need for inotropes/vasopressors was observed in 119 patients; 104 (87.4%) had qSOFA ≥2, and 117 (98.3%) had NEWS2 ≥5. Among those not requiring inotropes (N=36), 35 (97.2%) had qSOFA ≥2 and 25 (69.4%) had NEWS2 ≥5. Mechanical ventilation was required in 66 patients; 63 (95.5%) had qSOFA ≥2, and all 66 (100%) had NEWS2 ≥5. Among 89 patients not ventilated, 76 (85.3%) had qSOFA ≥2, and 76 (85.3%) had NEWS2 ≥5. Prolonged ICU stay occurred in 33 patients; 31 (93.9%) had qSOFA ≥2, and 32 (96.9%) had NEWS2 ≥5. Among 122 patients without prolonged ICU stay, 108 (88.5%) had qSOFA ≥2 and 110 (90.2%) had NEWS2 ≥5.

**Table 2:** Association between qSOFA and NEWS score with outcomes Values represent the number of patients, with percentages enclosed in parentheses. Total number of patients N = 155. qSOFA, quick Sequential Organ Failure Assessment; NEWS, National Early Warning Score.

Outcomes	qSOFA score		NEWS2 score	
	≥2	<2	≥5	<5
First 7-day mortality				
Yes (N=29)	29 (100%)	0 (0%)	29 (100%)	0 (0%)
No (N=126)	110 (87.3%)	16 (12.7%)	113 (89.6%)	13 (10.3%)
Need for inotropes/vasopressor				
Yes (N=119)	104 (87.4%)	15 (12.6%)	117 (98.3%)	2 (1.6%)
No (N=36)	35 (97.2%)	1 (2.8%)	25 (69.4%)	11 (42.3%)
Need for mechanical ventilation				
Yes (N=66)	63 (95.5%)	3 (4.5%)	66 (100%)	0 (0%)
No (N=89)	76 (85.3%)	13 (15.6%)	76 (85.3%)	13 (15.6%)
Prolonged ICU stay				
Yes (N=33)	31 (93.9%)	2 (6.1%)	32 (96.9%)	1 (3.03%)
No (N=122)	108 (88.5%)	14 (11.5%)	110 (90.16%)	12 (9.83%)

Both qSOFA and NEWS2 demonstrated excellent sensitivity and negative predictive value for predicting seven-day mortality, but had low specificity and positive predictive value. NEWS2 generally showed better specificity and negative predictive value compared to qSOFA for outcomes including mechanical ventilation, vasopressor or inotrope requirement, and prolonged ICU stay. At the same time, qSOFA had a slightly higher positive predictive value in some instances. Overall, NEWS2 performed better than qSOFA in predicting most clinical outcomes (Table 3).

**Table 3:** Diagnostic accuracy of qSOFA and NEWS2 for predicting outcomes PPV, positive predictive value; NPV, negative predictive value; qSOFA, quick Sequential Organ Failure Assessment; NEWS2, National Early Warning Score 2.

Diagnostic accuracy	95% CI	
	qSOFA	NEWS2
Mortality		
Sensitivity	1.00 (0.88, 1.00)	1.00 (0.88, 1.00)
Specificity	0.27 (0.20, 0.35)	0.25 (0.18, 0.33)
PPV	0.21 (0.14, 0.29)	0.20 (0.14, 0.28)
NPV	1.00 (0.91, 1.00)	1.00 (0.91, 1.00)
Mechanical ventilation		
Specificity	0.95 (0.87, 0.99)	1.00 (0.88, 1.00)
Specificity	0.33 (0.25, 0.43)	0.25 (0.18, 0.33)
PPV	0.45 (0.37, 0.54)	0.20 (0.14, 0.28)
NPV	0.93 (0.80, 0.98)	1.00 (0.91, 1.00)
Vasopressor/inotrope need		
Specificity	0.87 (0.80, 0.93)	0.98 (0.94, 1.00)
Specificity	0.43 (0.30, 0.56)	0.59 (0.46, 0.71)
PPV	0.75 (0.67, 0.82)	0.82 (0.75, 0.88)
NPV	0.63 (0.47, 0.78)	0.95 (0.82, 0.99)
Prolonged ICU stay		
Specificity	0.94 (0.80, 0.99)	0.97 (0.84, 1.00)
Specificity	0.27 (0.20, 0.34)	0.25 (0.18, 0.33)
PPV	0.22 (0.16, 0.30)	0.23 (0.16, 0.30)
NPV	0.95 (0.83, 0.99)	0.97 (0.86, 1.00)

The AUC analysis showed that NEWS2 performed better than qSOFA in predicting all measured outcomes, suggesting that NEWS2 has better overall predictive value for mortality, organ support needs, and ICU outcomes (Table 4).

**Table 4:** Area under the curve for qSOFA and NEWS2 scores in predicting outcomes qSOFA, quick Sequential Organ Failure Assessment; NEWS2, National Early Warning Score 2.

Test result variable(s)	Area under the curve	Significance	95% CI	
			Lower bound	Upper bound
Mortality				
qSOFA score	0.736	<0.001	0.644	0.828
NEWS2 score	0.949	<0.001	0.905	0.993
Vasopressor/inotrope need				
qSOFA score	0.780	<0.001	0.629	0.787
NEWS2 score	0.911	<0.001	0.865	0.958
Mechanical ventilation				
qSOFA score	0.713	<0.001	0.637	0.789
NEWS2 score	0.883	<0.001	0.834	0.932
Prolonged ICU stay				
qSOFA score	0.6	0.072	0.504	0.697
NEWS2 score	0.707	<0.001	0.637	0.788

**Figure 2:**
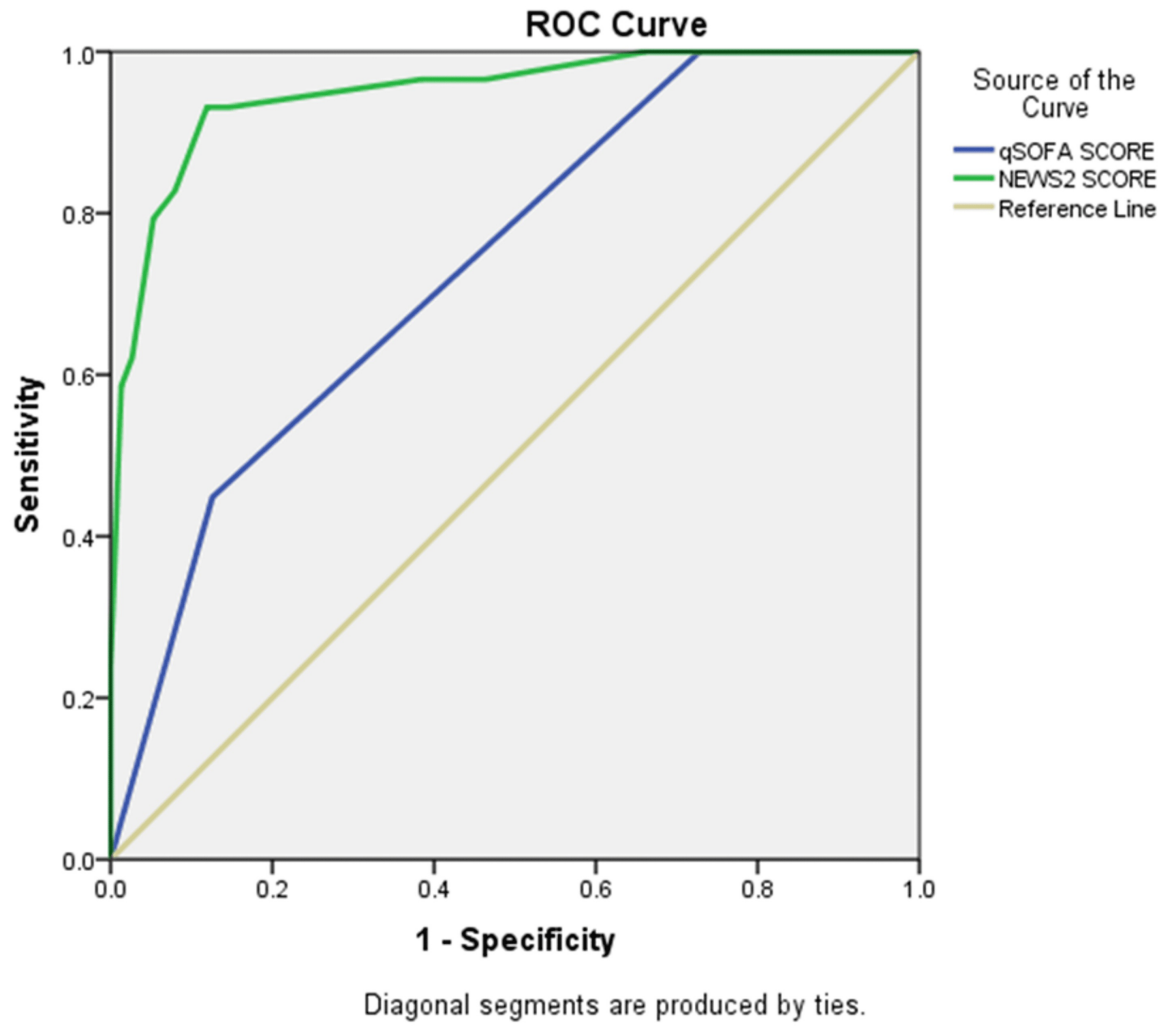
ROC curve for qSOFA and NEWS2 in predicting mortality among patients with sepsis ROC, receiver operating characteristic; qSOFA, quick Sequential Organ Failure Assessment; NEWS2, National Early Warning Score 2.

Figure 2 shows that the NEWS2 score accurately predicts mortality in sepsis patients better than qSOFA. The ROC curve for NEWS2 lies above that of qSOFA and farther from the diagonal reference line, indicating stronger predictive ability. This suggests that NEWS2 has higher sensitivity and specificity across various thresholds, while qSOFA shows lower accuracy. Overall, NEWS2 appears to be a more reliable predictor of mortality in this patient group.

**Figure 3:**
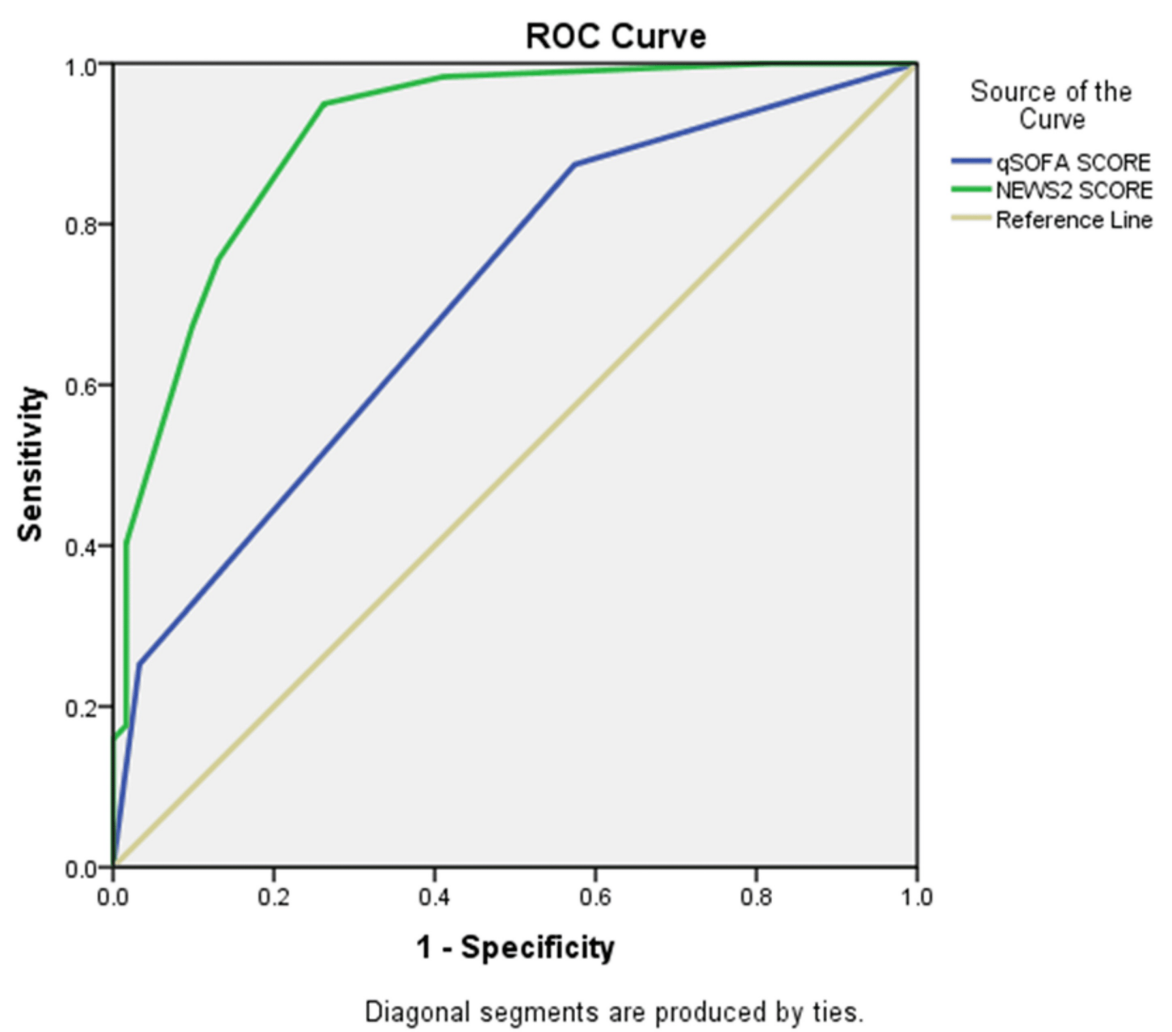
ROC curve for qSOFA and NEWS2 in predicting the need for inotropes or vasopressors among patients with sepsis ROC, receiver operating characteristic; qSOFA, quick Sequential Organ Failure Assessment; NEWS2, National Early Warning Score 2.

Figure 3 demonstrates that the NEWS2 score has superior predictive ability compared to qSOFA for identifying patients with sepsis who require inotropes or vasopressors. The ROC curve for NEWS2 lies consistently above that of qSOFA and remains farther from the diagonal reference line, indicating better overall discrimination. This suggests that NEWS2 provides higher sensitivity and specificity across various cutoff points, whereas qSOFA shows comparatively lower accuracy. Overall, NEWS2 appears to be a more effective tool for predicting the need for hemodynamic support in this study population.

**Figure 4:**
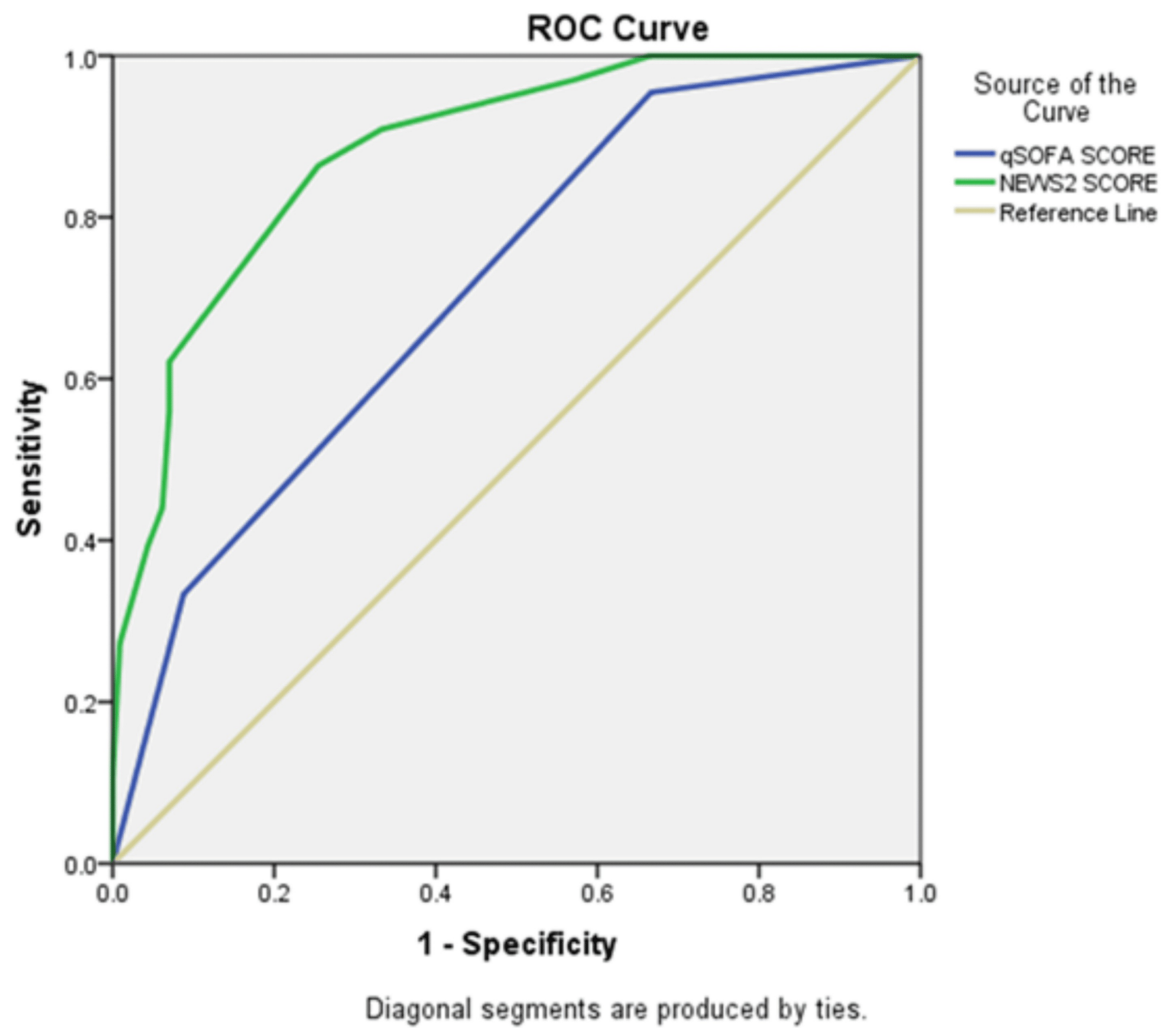
ROC curve for qSOFA and NEWS2 in predicting the need for mechanical ventilation among patients with sepsis ROC, receiver operating characteristic; qSOFA, quick Sequential Organ Failure Assessment; NEWS2, National Early Warning Score 2.

Figure 4 shows that NEWS2 performs better than qSOFA in predicting mechanical ventilation requirements among patients with sepsis. The ROC curve for NEWS2 remains consistently above that of qSOFA and is farther from the diagonal reference line, indicating stronger discriminative ability. This suggests that NEWS2 achieves higher sensitivity and specificity across different cutoff values, while qSOFA provides comparatively lower predictive accuracy. Overall, NEWS2 appears to be a more reliable scoring system for predicting the need for mechanical ventilation in this study population.

**Figure 5:**
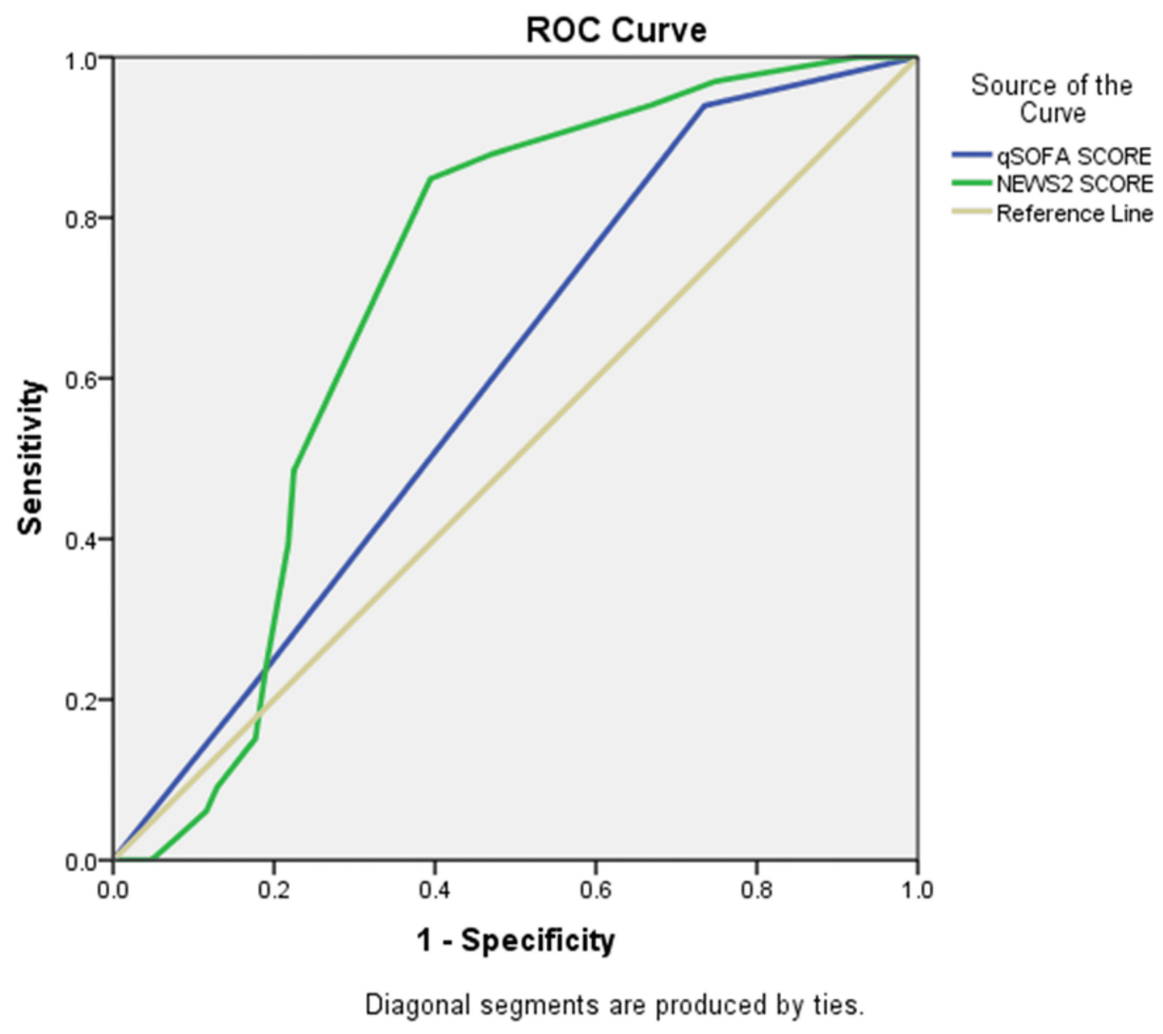
ROC curve for qSOFA and NEWS2 in predicting prolonged ICU stay among patients with sepsis ROC, receiver operating characteristic; qSOFA, quick Sequential Organ Failure Assessment; NEWS2, National Early Warning Score 2.

Figure 5 shows that NEWS2 has better discriminative ability than qSOFA for predicting prolonged ICU stay among patients with sepsis. The ROC curve for NEWS2 lies above that of qSOFA across most threshold values and is further from the diagonal reference line, indicating improved sensitivity and specificity. In contrast, qSOFA shows comparatively lower predictive accuracy, as its curve remains closer to the reference line. Overall, these findings suggest that NEWS2 is a more reliable predictor of prolonged ICU stay in this study population.

## Discussion

The results showed that, compared with qSOFA, NEWS2 had better predictive ability for prolonged ICU stays, inotropic support requirements, mechanical ventilation requirements, and seven-day in-hospital mortality. The study population was slightly more men (53%), with a mean age of 65. In 68% of patients, type 2 diabetes mellitus was the most common comorbidity.

Significant efforts have been made over the last three decades to enhance sepsis prognostication, risk classification, and early detection. Sepsis is still a major cause of in-hospital death globally, even with improvements in critical care and antibiotic therapy. In critically ill patients, severity score systems are frequently employed to predict clinical outcomes and stratify risk [16].

The qSOFA score was developed to facilitate rapid bedside identification of patients with suspected infection who are at increased risk of adverse outcomes, particularly short-term mortality. Since its introduction, qSOFA has been externally validated and compared with earlier tools such as SIRS for mortality prediction. George et al. [16] reported that SIRS demonstrated a sensitivity of 89% for predicting mortality, compared with 80% for qSOFA. Similarly, Henning et al. [17] observed that SIRS had higher sensitivity (83%) than qSOFA (52%) for mortality prediction. These findings suggest that although qSOFA is simple and practical, its sensitivity may be suboptimal in certain clinical settings.

The Royal College of Physicians developed the NEWS in 2012 to identify patients, particularly those with sepsis, at risk of clinical deterioration [10]. With a larger AUROC, Mellhammar et al. [18] showed that NEWS2 outperformed qSOFA in identifying patients with infection-related organ failure, infection-associated death, and ICU admission. Our results supported these findings, as NEWS2 outperformed qSOFA in AUROC across all evaluated outcomes.

Churpek et al. [19] reported that qSOFA had an AUROC of 0.69 (95% CI 0.67-0.70), which was lower than that of NEWS (0.77) and Modified Early Warning Score (MEWS) (0.73) for predicting clinical deterioration. Brink et al. [14] found that NEWS was the most accurate tool for predicting 10- and 30-day mortality among emergency department patients with sepsis, followed by SIRS and qSOFA. Similarly, Verma et al. [20] reported that NEWS2 outperformed qSOFA in predicting adverse outcomes in patients with sepsis. These observations align with the results of the present study.

The NEWS2 scoring system employs a comprehensive physiological approach, incorporating multiple vital signs, including respiratory rate, oxygen saturation, systolic blood pressure, heart rate, level of consciousness or new-onset confusion, supplemental oxygen requirement, and temperature [21,22]. This multidimensional assessment likely contributes to its enhanced predictive accuracy. In the present study, both NEWS2 and qSOFA were significantly associated with adverse outcomes; however, NEWS2 demonstrated greater sensitivity in predicting seven-day mortality, mechanical ventilation, and inotropic support. Both scoring systems showed comparable sensitivity for predicting prolonged ICU stay.

In many emergency department settings, vital signs are often recorded only once during triage [23]. Given that clinical status may evolve rapidly in patients with sepsis, reliance on a single set of physiological measurements may limit the predictive accuracy of early warning systems. Nevertheless, NEWS2 remains clinically valuable, particularly when scores are ≥5, which may indicate an increased risk of deterioration [24].

A systematic review of patients with suspected infection outside the ICU reported that a NEWS score ≥5 predicted mortality with a pooled sensitivity and specificity of 0.80 and an AUROC of 0.80 (95% CI 0.71-0.86) [25]. These findings were comparable to those observed in the present study, in which NEWS2 demonstrated excellent discrimination for mortality prediction (AUROC 0.949; 95% CI 0.905-0.993), whereas qSOFA showed moderate discrimination (AUROC 0.736; 95% CI 0.644-0.828). Additionally, a large study involving over 91,000 emergency department visits across two hospitals in England reported high predictive accuracy (AUROC >0.90) for NEWS scores ≥5, indicating a favorable balance between sensitivity and specificity [26].

The clinical value of a risk stratification score depends largely on the outcome measures selected. Many prior studies on qSOFA focused exclusively on mortality [27]. In contrast, our study evaluated additional outcomes, including the need for mechanical ventilation, the use of inotropes/vasopressors, and a prolonged ICU stay. Focusing solely on mortality can overlook clinically significant deterioration, and with increasing awareness of sepsis’s long-term impact, broader outcome measures are essential. Apart from mortality, prolonged ICU stay and the need for advanced support such as mechanical ventilation or vasopressors determine adverse outcomes and influence long-term patient morbidity.

Limitations and future scope

The limitations of this study include its single-center observational design and relatively modest sample size. As a prospective observational cohort study, although temporality was maintained, the design remains susceptible to inherent limitations such as selection bias and the inability to establish causal relationships. The relatively small sample size may also limit the generalizability of the findings and affect the precision and reliability of the estimated diagnostic performance measures.

In addition, potential sources of bias, such as spectrum bias, may be present, as the study population was limited to ICU-admitted patients and may not reflect the full clinical spectrum of sepsis. Verification bias may also have influenced the results, as outcomes were assessed within a hospitalized cohort and not all patients underwent uniform external validation. Furthermore, review bias cannot be completely excluded, as clinical assessment and score calculation were performed by the investigator, potentially introducing observer-related variability.

Another limitation is that qSOFA and NEWS2 scores were calculated only at the time of ICU admission, and serial measurements or earlier assessment at hospital presentation were not performed, which might have provided additional insights into dynamic changes in patient status and improved prognostic accuracy.

Despite these limitations, the findings highlight the potential of NEWS2 as a more effective tool than qSOFA for predicting adverse outcomes in hospitalized patients with sepsis. This study provides a foundation for future research in the Indian context, where simple bedside clinical tools often have practical advantages over expensive technologies and biomarkers. Large-scale studies validating newer tools for sepsis diagnosis and outcome prediction are warranted to further strengthen these findings.

## Conclusions

The present study demonstrates that NEWS2 outperforms qSOFA in predicting in-hospital mortality among patients with sepsis, with superior overall prognostic accuracy. NEWS2 also showed better performance in identifying patients at risk of requiring mechanical ventilation, vasopressor support, and prolonged ICU stay. While qSOFA remains a simple bedside tool, its role is more suited for prognostication rather than early screening due to its limited sensitivity. In contrast, NEWS2 appears to be a more reliable tool for both early detection of clinical deterioration and risk stratification in hospitalized patients with sepsis.
